# High frequency UV–Vis sensors estimate error in riverine dissolved organic carbon load estimates from grab sampling

**DOI:** 10.1007/s10661-022-10515-9

**Published:** 2022-09-26

**Authors:** J. P. Ritson, O. Kennedy-Blundell, J. Croft, M. R. Templeton, C. E. Hawkins, J. M. Clark, M. G. Evans, R. E. Brazier, D. Smith, N. J. D. Graham

**Affiliations:** 1 School of Environment, Education and Development, The University of Manchester, Oxford Rd, Manchester, M13 9PL UK; 2 Department of Civil and Environmental Engineering, Imperial College London, South Kensington, London, SW7 2AZ UK; 3 Department of Geography and Environmental Science, University of Reading, Whiteknights, PO box 227, Reading, RG6 6AB UK; 4 Centre for Resilience in Environment, Water and Waste, Geography, University of Exeter, Exeter, EX44RJ UK; 5 South West Water, Peninsula House, Rydon Lane, Exeter, EX2 7HR UK

**Keywords:** Dissolved organic carbon, Load estimate, UV–Visible absorbance, In-river sensors, Water quality monitoring

## Abstract

High frequency ultraviolet – visible (UV–VIS) sensors offer a way of improving dissolved organic carbon (DOC) load estimates in rivers as they can be calibrated to DOC concentration. This is an improvement on periodic grab sampling, or the use of pumped sampling systems which store samples in-field before collection. We hypothesised that the move to high frequency measurements would increase the load estimate based on grab sampling due to systemic under-sampling of high flows. To test our hypotheses, we calibrated two sensors in contrasting catchments (Exe and Bow Brook, UK) against weekly grab sampled DOC measurements and then created an hourly time series of DOC for the two sites. Taking this measurement as a ‘true’ value of DOC load, we simulated 1,000 grab sampling campaigns at weekly, fortnightly and monthly frequency to understand the likely distribution of load and error estimates. We also performed an analysis of daily grab samples collected using a pumped storage sampling system with weekly collection. Our results show that: a) grab sampling systemically underestimates DOC loads and gives positively skewed distributions of results, b) this under-estimation and positive skew decreases with increasing sampling frequency, c) commonly used estimates of error in the load value are also systemically lowered by the oversampling of low, stable flows due to their dependence on the variance in the flow-weighted mean concentration, and d) that pumped storage systems may lead to under-estimation of DOC and over estimation of specific ultra-violet absorbance (SUVA), a proxy for aromaticity, due to biodegradation during storage.

**Supplementary Information:**

The online version contains supplementary material available at 10.1007/s10661-022-10515-9.

## Introduction

An accurate estimate of dissolved organic carbon (DOC) flux, that is the total amount of DOC exported per unit area in a given period, is crucial to understanding the movement of organic carbon from soils to rivers and into oceans, affecting the global carbon cycle (Cole et al., 2007). DOC concentrations in rivers have become a focus for research because of the increases in concentration noted in acid sensitive catchments across Europe and North America in recent decades (e.g. Monteith et al., 2007). High DOC exports are typically associated with organic soils and wetlands areas in the catchment, although other areas like woodlands have also been associated as a source of DOC in rivers (Ritson et al., 2019). As well as global carbon budgets, rising DOC concentrations are an issue for drinking water treatment where it can create increased operational costs to remove it (Ødegaard et al., 2010), and treated water quality problems (e.g. disinfection byproduct formation, microbial growth (Ritson et al., 2014)). Consequently, decreases in DOC concentration can be seen as one component of success in catchment management programmes (Alderson et al., 2019, Grand-Clement et al., 2013).

Despite knowledge that at some sites approximately 50% of DOC flux is accounted for in the top 10% of flows (Clark et al., 2007), inclusion of storm sampling or high frequency sampling in monitoring campaigns has been limited for practical reasons. For example, the UK Upland Waters Monitoring Network, running since 1988, samples rivers for DOC at monthly frequency and lakes quarterly (Warren et al., 1986). Typically, monthly, fortnightly or weekly sampling is used to estimate a flow-weighted mean DOC concentration which is then multiplied by the mean discharge for the time period to estimate load (e.g. Method 5 in Walling & Webb, 1985). The error in this load estimate is then assessed using the variance in the flow-weighted mean concentration (Hope et al., 1997). Recent examples of this technique being employed to estimate DOC loads or fluxes include Arízaga-Idrovo et al., 2022; Gaffney et al., 2020, Pérez-Rodríguez & Biester, 2022, Regensburg et al. 2022; Rosset et al., 2019 and our own work in Ritson et al., 2019.

This low frequency manual ‘grab’ sampling means baseflow conditions are likely to be over-sampled and periods of high flows may be missed, meaning the relationship between DOC and flow is only understood for a small section of the hydrograph. This is crucial in areas where export of DOC from key source areas, like peatlands, mainly occurs at high flow (Clark et al., 2007). Furthermore, the over-sampling of baseflows is likely to lead to low variance in the flow-weighted mean concentration, giving artificially low error values for the load estimate. Where hourly or daily sampling is employed, this is often facilitated using automated pump-sampling systems which then store the unfiltered sample at ambient temperatures until collection. Biodegradation of DOC may occur for some samples stored in the field, leading to artificially lower DOC values when analysed in the laboratory. Some studies, however, return samples to the laboratory within hours for stabilisation (e.g. Glendell and Brazier, 2014)) and samplers with built-in refrigeration are becoming more common and may limit this problem.

High frequency ultraviolet–visible (UV–Vis) sensors are becoming more common for estimating DOC fluxes as they offer a method of estimating DOC concentration at a resolution that can overcome the issues of manual and automatic sampling (Grayson & Holden, 2016; O’Driscoll et al., 2018; Ruhala & Zarnetske, 2016). These higher resolution data, collected at intervals of minutes or hours, offer insight into, for example, the hysteresis between DOC concentration and flow. This is critical in developing accurate estimates of load as DOC concentration can both increase and decrease with flow depending on the characteristics of the catchment under study and the scale of the high flow event (Clark et al., 2007). However, sensors have reported issues associated with drift and the need for maintenance and site-specific calibration. Wider evaluation of their use is needed through comparison with grab and automatic pump sampling.

Here, we compare the efficacy of UV–Vis sensors in contrasting environments. We employed two scanning in-river UV–Vis sensors to estimate DOC concentration in two different upland and lowland catchments, the Exe and Bow Brook, UK, at hourly resolution to understand the difference in load and error estimates when the DOC-flow relationship is characterised for the entire hydrograph, rather than by periodic grab sampling. We hypothesised that, a) the load estimate will be higher from high resolution sampling, though the extent of this will depend on catchment DOC transport processes, specifically the predominance of dilution versus flushing of DOC sources with increases in flow, b) that differences in the load estimate will be greater in smaller, flashier catchments where short, high flow events may be missed by grab sampling, and c) the error based on time of sampling will be greater than that based on the variance in the flow-weighted mean concentration as baseflow conditions will be oversampled. In a related experiment, we also tested the stability of DOC samples in un-refrigerated automatic samplers to assess if this offers a viable method of increasing sampling frequency.

## Materials and methods

### Sites and instrumentation

The Exe catchment is 600.9 km^2^ and contains a mixture of blanket bog and acid grassland in the headwaters, with an increasing proportion of woodland and agricultural land further down, all of which have been shown to significantly influence DOC concentrations in the tributaries (Ritson et al., 2019). Further details of the catchment at the sampling point can be found in Ritson et al. (2019).

On the Exe we installed a TriOS OPUS UV–Vis Spectral Analyser at an offtake for a water treatment works near Brampford Speke for the period 1/10/2019 to 16/07/2020. The instrument was the same as previously used for analysis of DOC samples upstream, giving confidence in performance (Grand-Clement et al., 2014). Our intention to monitor for 12 months was cut short first by staff illness and then the Covid-19 pandemic so we present results from nine months of data. The instrument took a scan from 200–800 nm at 2.2 nm resolution on an hourly basis with a wiper cleaning of the sensor window before each measurement. The instrument was visited monthly for cleaning and to perform a blank measurement against ultrapure water. We obtained flow data for the site using the National River Flow Archive station ‘Exe at Thorverton (45,001)’ which is approximately 6.05 km upstream. For the calibration dataset we used an existing monitoring programme performed by South West Water at the site which collected weekly samples. DOC was measured as non-purgeable organic carbon (NPOC) on samples filtered to 0.45 µm and analysed by a Shimadzu TOC-L thermal combustion instrument.

The Bow Brook is a much smaller catchment than the Exe with an area of 40.4 km^2^ and contains a much greater proportion of lowland agricultural land use as well as inputs from nearby sewage treatment works. On the Bow Brook (51.322583, -1.029167) we installed the same type of instrument in a remote monitoring station developed by the Environment Agency for the period 08/09/17 to 09/09/18. This involved a pump sampling system whereby each hour a sample of water was pumped from the river and measured by the Trios system with the aim of reducing fouling by minimising the contact time between sensor and water. The sensor was cleaned weekly due to greater fouling observed at this site. A Sontek IQ flow gauge that uses Doppler technology was used to measure river flow at the same point that the samples were taken.

Bow Brook calibration samples were collected weekly and filtered to 0.7 µm and analysed using a UV-persulphate method on a Shimadzu TOC-L. Note, due to the differences in analytical methodology between sites, specifically the difference in filter paper size and instrument methodology, we do not compare absolute DOC values between them. Daily grab samples collected during summer were also analysed to assess the utility of automated sampling systems to offer higher frequency grab sampling. Daily samples were taken using an ISCO 6712 autosampler which were then collected weekly, meaning the samples were between 0 and 7 days old at the time of collection. A UV-absorbance measurement was also taken in the laboratory on samples filtered to 0.7 µm using a Hellma Analytics High Precision synthetic quartz glass cuvette and a Jenway 7315 Spectrophotometer. Specific UV absorbance (SUVA) was calculated as the absorbance at 254 nm in units of m^−1^ divided by the NPOC content (mgC l^−1^). Water temperature was measured hourly using a multi-parameter Sonde (YSI 6600 V2—2). The dataset and further information on the Bow Brook can be found in Hawkins et al. (2019).

### DOC and UV–Vis calibration

At the Bow Brook site 25 weekly (18.09.2017 – 11.04.2018) DOC grab samples were used to calibrate the UV–Vis sensor outputs to the river DOC concentration. This sample covered 87.7% of the flow conditions observed during the sampling period with 10.7% lower than the calibration point and 1.6% higher. Prior to any analysis, spectra were quality checked and discarded if the 200/400 nm ratio was < 2, as these samples were deemed to be affected by sensor fouling. Sensor fouling causes an increase in absorbance at all wavelengths, meaning the spectra appear flat, rather than showing a large peak at ~ 200 nm and then a rapid decline which is characteristic of river water samples due to absorbance from nitrate and aromatic carbon from ~ 200 – 260 nm, with lower absorbance in the coloured region (Weishaar et al., 2003). Our choice of threshold value was based on visual assessment of the spectra which showed rapid fouling when it did occur and led to flattening of the spectra. The retention of some partially fouled data may have contributed to the poorer correlation coefficient at the Bow Brook site. As the sensor was cleaned weekly, this is unlikely to affect many data points.

All sensor data were then analysed using the R ‘PLS’ package for partial least square analysis, with cross validation steps to prevent any overfitting of the statistical model. 21 components were selected from 248 wavelengths following initial analysis and prior to cross validation steps. Following cross validation, seven components were selected with variable importance in projection scores > 1 (Li et al., 2020). This chosen number of components provided a strong degree of explanatory power without overfitting the model, achieving a cross validation correlation of 0.55. This was an improvement over single wavelength models which achieved a maximum correlation to the DOC data of 0.31 at 400 nm. Calibration sampling covered 98.27% of the modelled DOC conditions observed during the sensor monitoring period, with 1.73% higher than the maximum calibration DOC concentration and no points lower than the minimum.

For the Exe we used 26 weekly DOC grab samples to calibrate the UV–Vis sensor output to the river DOC concentration. This sample covered 91.4% of the flow conditions observed during the sampling period with 5.9% lower than the calibration point and 2.7% higher. The PLS achieved a cross validation correlation of 0.89 from a three-component model. The number of components was selected using the mean square error with increasing numbers of components and visually inspecting the resulting DOC time series for noisy, overfitted data. This was an improvement over single wavelength models which achieved a maximum correlation to the DOC data of 0.54 at 230 nm. The calibration sample covered 97.74% of the modelled DOC values with 1.77% lower than the minimum calibration condition and 0.49% higher than the highest calibration point.

A better correlation between the UV–Vis spectra and DOC was achieved on the Exe compared to the Bow Brook. This is likely due to the differing characteristics of the DOC at the two sites with the Exe being influenced by a high proportion of organic rich soils, producing DOC which is highly coloured with strong absorbance in the UV range due to greater aromaticity. Bow Brook, on the other hand, contains mainly lowland agriculture and may also be influenced by effluent from sewage treatment works within the catchment, potentially meaning a greater proportion of aliphatic DOC which does not absorb in the UV range, leading to poorer correlations. Yang et al., 2021, in their assessment of the UV persulphate method, found that it a humic acid standard gave incomplete oxidation, leading to an underestimation of DOC compared to citric acid and tannic acid standards. We therefore suggest that the DOC values from Bow Brook are likely correct, but that the correlation is poor as the DOC present does not fully absorb in the UV. The differences in correlations may also be explained by the differing filtration and analytical procedure between the two sites.

### Simulation of grab sampling programmes

Once we had a time series of hourly DOC for the two sites, we calculated the total load for the period based on the hourly DOC and flow measurements and took this to be a ‘true’ value for comparison of different sampling regimes against. While there will still be error in this estimate due to imperfect calibration, these offer a best estimate for which to compare against. Our models achieved a cross validation correlation of 0.89 in the Exe and 0.54 in Bow Brook, suggesting much greater confidence in the validity of the assumption that the UV data gives a ‘true’ value of load in the Exe than in Bow Brook.

We then simulated grab sampling of different frequencies using a random number generator (Excel, Microsoft Corporation) to pick a measurement at weekly, fortnightly and monthly frequency for the period of interest and then calculated a load and error estimate using the flow-weighted mean DOC concentration (Method 5 in Walling & Webb, 1985) and the variance in the flow-weighted mean concentration (Hope et al., 1997), respectively. This was repeated 1,000 times for each sampling frequency to create a distribution of load and error estimates for each site and frequency. These distributions were then analysed for skew and kurtosis in SPSS (IBM).

Subsequently, we calculated a 95% confidence interval for our load estimate based on the difference between each simulated grab sample and the ‘true’ value based on hourly data, expressed as a percentage. As our simulated grab sampling data were not normally distributed we used Chebyshev’s Theorem to assess the range that 95% of results in the distribution fell within. We used this value to quantify the spread of likely results from grab sampling, suggesting the error associated with the incomplete sampling of the hydrograph.

## Results

### Sensor fouling

**Fig. 1:**
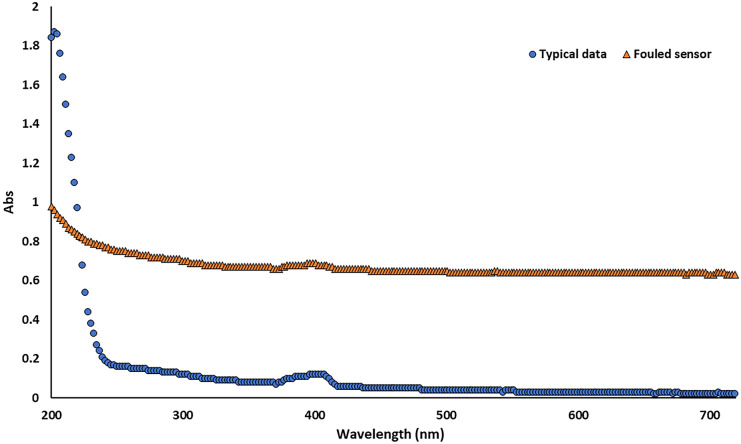
UV–Vis sensor data from the Bow Brook calibration dataset collected when the sensor was clean and when it was fouled

Figure 1 shows the difference in spectra between typical data and that which has been affected by sensor fouling. Both UV–Vis scans were taken in October 2017 on the date of calibration visits, so laboratory DOC data are also available. The ‘typical data’ are representative of the bulk of the dataset, whereas the ‘fouled sensor’ shows a flat line which does not approach zero even at the highest wavelengths and has a very low nitrate peak. We take this to be indicative of biofilm growth affecting absorbance at all wavelengths. DOC concentration in the river at the times of the scans was similar at 8.8 mg l ^−1^ for the clean sample and 8.5 mg l ^−1^ for the fouled sample. Based on this, we discarded data from the calibration dataset where sensor fouling was an issue.

### Comparison of load and error estimates

Table 1 shows the difference in distributions of the 1,000 simulated sample events for the different frequencies at our two sites. Increasing the grab sampling frequency reduces the difference in median value from the ‘true’ value of load as well as the skew and kurtosis of the distribution of results. For monthly and fortnightly sampling, our estimate of error based on the time of sampling was an order of magnitude higher than that of the commonly used method of Hope et al. (1997). We do not present absolute values for the calculated load as they are not comparable between sites due to the difference in analytical methodology and because our main interest is understanding the distribution of expected results from differing grab sampling frequencies.

**Table 1:** Distributions of simulated sampling events for the Exe and Bow Brook catchments

		**Mean difference in median (%)**	**Skew**	**Kurtosis**	**Mean confidence interval Hope et al. method (%)**	**Confidence interval Chebyshev’s Theorem (%)**
**Exe**	Monthly	-4.53	0.82	0.83	± 6.41	± 41.1
	Fortnightly	-1.67	0.69	0.61	± 5.60	± 29.8
	Weekly	-1.09	0.45	0.01	± 2.38	± 20.1
**Bow Brook**	Monthly	-9.28	1.43	2.24	± 3.65	± 24.7
	Fortnightly	-8.39	0.94	0.63	± 3.28	± 16.7
	Weekly	-7.77	0.80	0.53	± 1.39	± 11.3

**Fig. 2:**
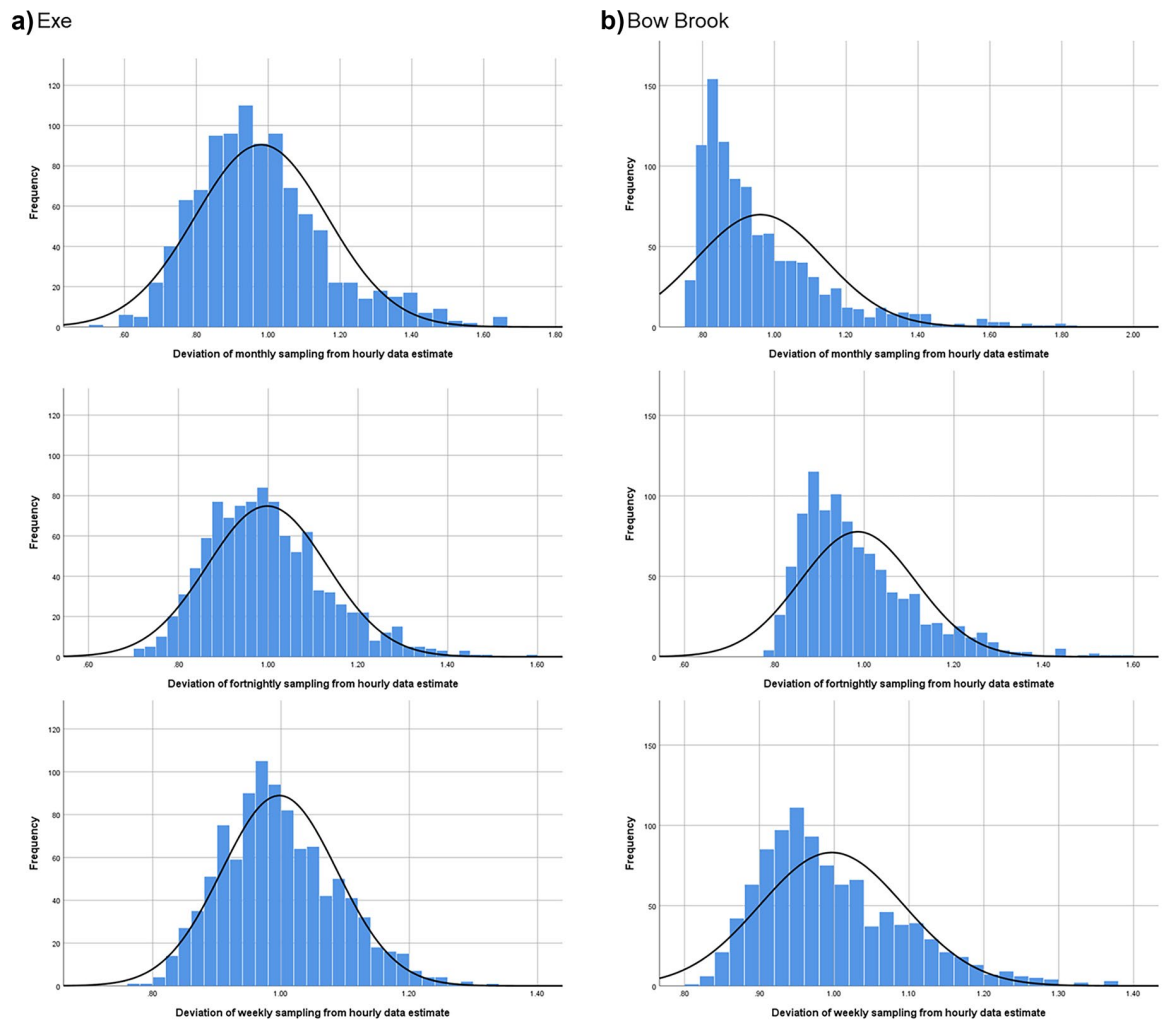
Distribution of simulated grab sampled events for **a** the Exe, and **b** Bow Brook. Data are presented as deviation from the estimate from hourly data as the absolute values between the sites are not comparable due to analytical differences

The distributions of results (Fig. 2) were significantly different for each sampling frequency on the Exe (Kruskall-Wallace test = 34.40, p < 0.001, df = 2) and the Bow Brook (Kruskall-Wallace test = 19.04, p < 0.001, df = 2). The much greater skew and kurtosis in the distribution of results at Bow Brook are likely explained by days in the dataset where no flow was recorded, meaning zero values for load where a simulated sampling event occurred on those days. This may also explain the lower confidence intervals compared to the Exe.

### Biodegradation of samples stored in the autosampler

To assess the possibility of biodegradation in samples collected and stored in an autosampler, we compared samples aged between 0 and 7 days over four sampling weeks during a period of stable flow from 25/09/2017 to 22/10/2017. During this period the daily mean flow averaged 0.205 ± 0.015 m^3^ s^−1,^ which was exceeded 68.9% of the time in the full annual record. Hourly water temperature during this period averaged 13.39 (range 11.19 – 15.39) ^o^C.

**Fig. 3:**
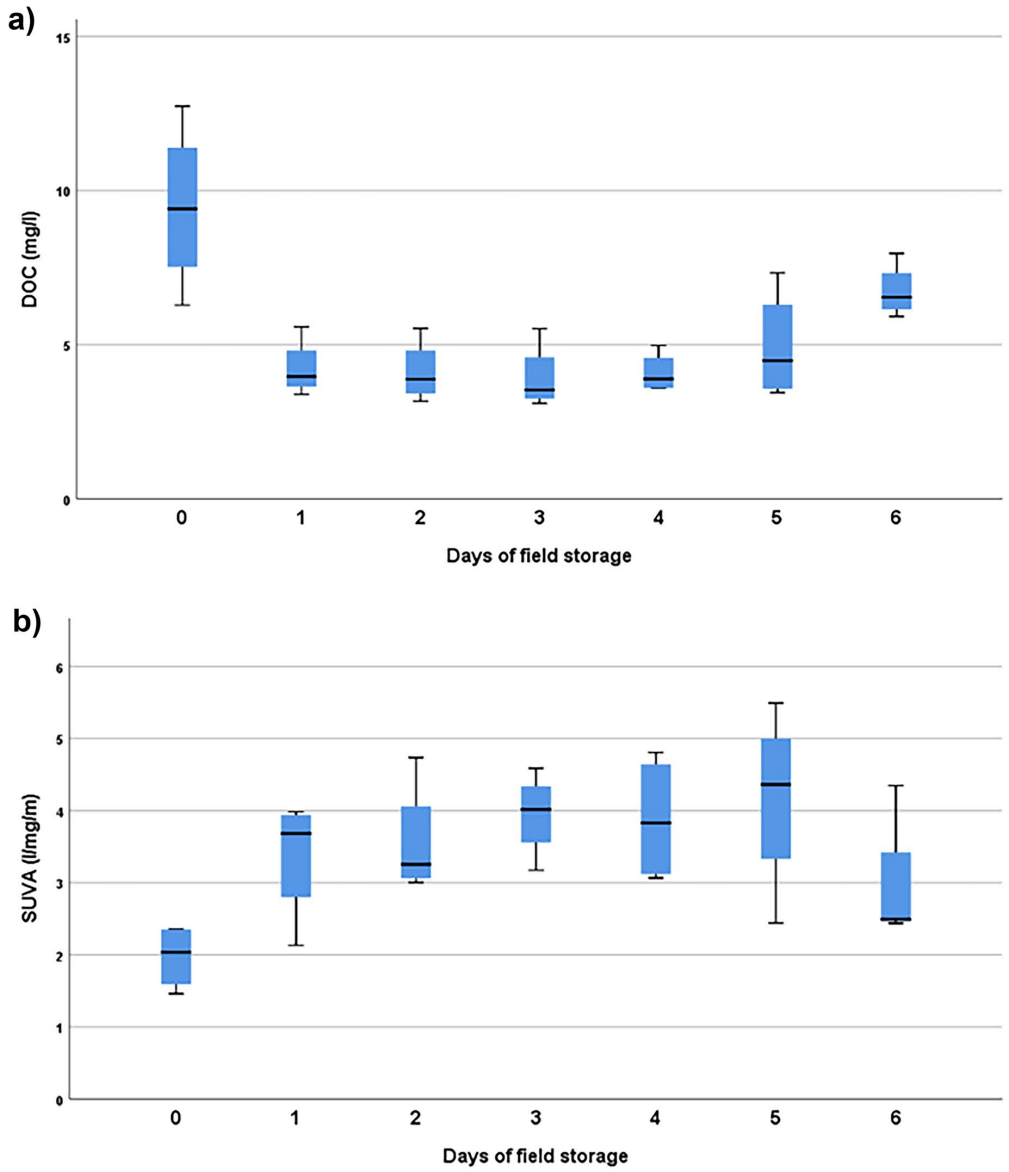
Differences in mean **a** DOC and **b** SUVA values for increasing duration of field storage at the Bow Brook site

We tested the effect of time since sampling on NPOC concentration and SUVA value using ANOVA with a log transformation applied to the SUVA data as it did not initially pass Levene’s test. This suggested that SUVA was significantly affected by time elapsed since sampling (F = 4.00, p = 0.08, df = 6), as was NPOC concentration (F = 8.05, p < 0.01, df = 6). No significant effect (F = 0.98, p = 0.46, df = 6) was detected for differences in UV–Vis sensor data at 254 nm for the same days, suggesting the changes in DOC and SUVA were only in the pumped samples, rather than general changes in river water quality. Over the four weeks, NPOC concentration averaged 9.46 mg l^−1^ in the samples collected the same day, versus 4.67 mg l^−1^ in the stored samples, whilst SUVA averaged 1.97 l mg^−1^ m^−1^ in the samples collected the same day, versus 3.64 l mg^−1^ m^−1^ in the stored samples. Figure 3 shows the change in DOC concentration and SUVA of the field-stored samples over time.

## Discussion

### Load and error estimate from hourly sensor data versus grab sampled data

Our results show a systematic underestimation of DOC load based on grab sampling results, hypothesis a), as the distributions are skewed towards lower values as high flow events are likely to be missed during sampling. Median load from the grab sampling frequencies is 95.5%, 98.3% and 98.9% that of the ‘true’ value derived from hourly DOC and flow data on the Exe. This may be a conservative estimate as our nine months of sampling missed the autumn period where typically high DOC loads are observed in this catchment (Ritson et al., 2019). Further underestimation of the true flux of DOC may arise from the use of the UV-persulphate method, which has been shown to incompletely oxidise humic sources of DOC Yang et al. (2021),

Although the improvement in accuracy between fortnightly and weekly sampling was small, the large reduction in skew and kurtosis (Table 1) suggests much lower likelihood of achieving a result in the tails of the distribution, and therefore more frequent sampling still provides a benefit in terms of increased precision. Our assessment of the deviation of results from the ‘true’ value of load suggests the error may be an order of magnitude greater than that estimated by commonly employed methods.

Whilst it is possible to theorise the under sampling of high flow events based on flow data alone, our time series of DOC during multiple high and low flow events over a 9 month period allows for more accurate quantification of this effect, given that the DOC-flow relationship can vary during high flow events (Clark et al., 2007) and can be dependent on temperature and transport pathways (Dawson et al., 2008). The use of hourly DOC measurements has further benefits in that it will likely capture diel variation in DOC concentrations, which can be significant (Westhorpe et al., 2012).

Comparing the Exe and the Bow Brook suggests hypothesis b) is supported, though with less confidence due to the poorer correlation, in that the smaller catchment which was prone to both high and low flow events had a greater difference in median result from the ‘true’ value, as well as greater skew and kurtosis. This is consistent with the river as a chemostat hypothesis (Creed et al., 2015), whereby the variation in river discharge and chemistry decreases as river order increases, and would suggest the underestimation in DOC load that our results show, is likely to be greatest in lower order rivers. This would give greater confidence in results from grab sampling at the tidal limits of rivers (e.g. Worrall et al., 2018) as compared to those in headwater streams, though extreme events such as typhoons may confound this (Liu et al., 2021).

The poorer performance of the calibration between DOC concentration and UV–Vis spectra in the Bow Brook catchment mean the results should be interpreted with more caution than the Exe. Our results suggest the UV–Vis technique may not be suitable for rivers with strong autochthonous DOC influence. With a cross validation correlation of 0.55, however, we interpret this correlation as moderately strong and therefore present these results as an estimate of the underestimation of DOC load in smaller, flashier catchments despite the molecular characteristics of the DOC not being optimal for UV–Vis.

To test hypothesis c), we compared the distribution in load values from our simulated sampling campaigns to the mean error calculated from the variance in the flow-weighted mean concentration for each sampling campaign. At all sampling frequencies the 95% confidence interval for our results is an order of magnitude greater than that estimated by normal methods. Many of the individual simulated sampling events had very low errors as calculated by the Hope et al. method and yet had very poor accuracy. This occurred when all or most of the samples in the simulated campaign fell during similar flow conditions, leading to a very low variance in the flow-weighted mean concentration, and thus error estimate, but still an inaccurate load estimate. We therefore suggest hypothesis c) is supported, as our distribution of results suggests a much larger error as the distribution of results from grab sampling has a high degree of kurtosis.

### Biodegradation during storage in an autosampler

One method of increasing grab sampling frequency is to use autosamplers to collect a daily sample from a river with the operator collecting the samples typically on a weekly basis. Our results show a significant decrease in DOC concentration as well as a significant increase in SUVA, used as an indicator of the aromaticity of the DOC (Weishaar et al., 2003), during this storage period of the samples in the autosampler. During this time the samples were unfiltered and at ambient temperatures and so they may have been subject to DOC concentration decreases through biodegradation, as well as increases via desorption of organic carbon from particulates.

We believe the approximate halving in DOC concentration and approximate doubling in SUVA between fresh and stored samples is consistent with biodegradation, with the preferential preservation of aromatic compounds. Indeed, given ambient temperatures in the south east UK at the particular time of year (not measured but inferred from water temperatures), the conditions in the autosampler were not too dissimilar to those used in biodegradable dissolved organic carbon (BDOC) analytical procedures, albeit without the addition of excess nutrients, which incubate at 20 °C for seven days (McDowell et al., 2006).

Our results draw into question DOC load estimates derived from autosamplers where the samples are stored in-field for longer than a day, particularly during summer when temperatures are higher. Datasets which use this method are likely to underestimate the true value of DOC concentration and over-estimate the aromaticity of the DOC, as measured by SUVA. Based on the characterisation of the factors affecting the rate of biodegradation of DOC performed by McDowell et al., (2006), we expect this to be particularly true where ambient temperatures and nutrient concentrations are high, storage times are long, or where the DOC in question is from a particularly labile source. Our previous work in the tributaries of the Exe suggested a range of BDOC values from 18 – 45% (Ritson et al., 2019), hinting at the potential scale of the impact of this issue, though a greater understanding of seasonal and hydrological variation in BDOC would be needed to attempt to correct for this effect.

### Limitations

Much of our study rests on the assumption that the hourly DOC-flow measurements represent a ‘true’ value of load with which to compare the grab sampling against. The validity of this assumption rests firstly on the accuracy of the flow measurements and secondly, on the UV-DOC calibration. We assume the flow data to be as accurate as is reasonably possible given our Exe data come from the UK National River Flow Archive and the Bow Brook data was generated using UK Environment Agency staff, methods, and equipment. In any case, grab sampling would use the same flow methods and thus the UV-DOC calibration seems the most likely source of error.

We have good confidence in the calibration procedure as, a) our calibration sample covers nearly all of the flow conditions observed during the monitoring period and very few modelled DOC values were outside the calibration window, and b) the good model achieved via PLS. The latter was less so for the Bow Brook and so these results must be treated with more caution than the Exe, though they show similar trends. A further source of error for comparisons between the two sites is the variable performance of the UV-persulphate method in achieving complete oxidation of DOC, and thus quantification (Yang et al., 2021). As the two sites have very different sources of DOC, this provides further reasoning for not comparing absolute values between the two. Finally, we present data from only two sites, meaning the applicability of these findings should be tested further on rivers with both differing hydrology and catchment DOC sources.

## Conclusions

Taken together, we believe our data show that both the absolute value of riverine DOC load and the estimate of error in this value were systematically underestimated, with implications for carbon cycle modelling, assessment of catchment management programmes as well as drinking water treatment. Moving from monthly to fortnightly and weekly sampling produces significantly different distributions of results with increased accuracy and precision from weekly sampling.

Using autosamplers with a weekly collection period has been shown to also lead to underestimation of DOC loads and overestimation of SUVA due to biodegradation during storage in the sampler. Biodegradation depends on DOC properties, nutrient availability and temperature, which vary between locations. We therefore recommend that an assessment of biodegradation of samples at field temperatures is carried out to determine if this method can be used to increase sampling frequency. Alternatively, methods to supress biological activity may be explored.

Scanning UV–Vis sensors offer a method of assessing in-river DOC concentration at high resolution and, via PLS methods, give better correlation with DOC concentration than single wavelength sensors. Our approach, however, is not without drawbacks as we were only able to achieve a strong correlation between DOC and UV–Vis spectra for one of our two sites. Whilst UV–Vis sensors show strong promise for rivers which have highly aromatic DOC, alternative techniques may be necessary for rivers where there is a greater proportion of non-UV absorbing DOC.

## Supplementary Information

Below is the link to the electronic supplementary material.

Supplementary file1 (XLSX 48 KB)

## Data Availability

Data will be made availably via a supplementary information file with the final manuscript. Some data (Bow Brook site) are already available via Hawkins et al. (2019), hosted by the NERC Environmental Information Data Centre.
